# Trial-by-trial modeling of electrophysiological signals during inverse Bayesian inference

**DOI:** 10.1186/1471-2202-15-S1-O8

**Published:** 2014-07-21

**Authors:** Antonio Kolossa, Bruno Kopp, Tim Fingscheidt

**Affiliations:** 1 Institute for Communications Technology, Technische Universität Braunschweig, Braunschweig, 38106, Germany; 2 Department of Neurology, Hannover Medical School, Hannover, 30625, Germany

## 

Empirical support for the Bayesian brain hypothesis, although of major theoretical importance for cognitive neuroscience, is surprisingly scarce. The literature still lacks definitive functional neuroimaging evidence that neural activities code and compute Bayesian probabilities. Here, we introduce a new experimental design to relate electrophysiological measures to Bayesian inference. Specifically, an urns-and-balls paradigm was used to study neural underpinnings of probabilistic inverse inference. Event-related potentials (ERPs) were recorded from human participants who performed the urns-and-balls paradigm, and computational modeling was conducted on trial-by-trial electrophysiological signals. Five computational models were compared with respect to their capacity to predict electrophysiological measures. One Bayesian model (BAY) was compared with another Bayesian model which takes potential effects of non-linear probability weighting into account (BAY_S_). A predictive surprise model (TOP_S_) of sequential probability revisions was derived from the Bayesian models. A comparison was made with two published models of surprise (DIF [1] and OST [2]).

**Figure 1:**
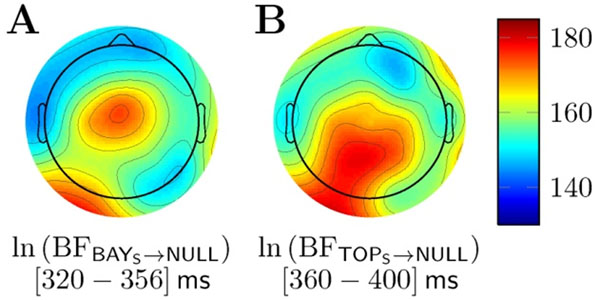
Scalp maps of averaged log-Bayes factors of models with non-linear probability weighting versus a null model. **A.** Bayesian surprise model (BAY_S_). **B.** Predictive surprise model (TOP_S_).

Subsets of the trial-by-trial electrophysiological signals were differentially sensitive to model predictors: The anteriorly distributed N250 was best fit by the DIF model, the BAY_S_ model provided the best fit to the anteriorly distributed P3a, whereas the posteriorly distributed P3b and Slow Wave were best fit by the TOP_S_ model. Figure 1 shows the model fit in log-Bayes factor [3] as scalp maps for the BAY_S_ and TOP_S_ models for P3a and P3b time windows, respectively. Table 1 summarizes the model comparison by translating the log-Bayes factors to posterior model probabilities [4] for all models and all ERPs at the respective time windows and electrodes. These results show that dissociable cortical activities code and compute different aspects of Bayesian updating. However, these activities might be best described as being Bayes optimal, implying that they reflect Bayesian inference, modulated by non-linear probability weighting, as originally conjectured by prospect theory [5,6].

**Table 1:** Posterior model probabilities.

	ERP waves and electrodes			
	
	N250	P3a	P3b	SW
Model	C4	FCz	Pz	O1
OST	0.02	< 0.01	< 0.01	< 0.01
DIF	0.66	< 0.01	< 0.01	< 0.01
TOP_S_	0.28	< 0.01	0.88	0.82
BAY	< 0.01	< 0.01	< 0.01	< 0.01
BAY_S_	0.04	0.99	0.12	0.18
